# Central circuit controlling thermoregulatory inversion and torpor-like state

**DOI:** 10.21203/rs.3.rs-2698203/v1

**Published:** 2023-03-24

**Authors:** Domenico Tupone, Shelby Hernan, Pierfrancesco Chiavetta, Shaun Morrison, Georgina Cano

**Affiliations:** Oregon Health and Science University; University of Pittsburgh; Oregon Health and Science University; Oregon Health & Science University; University of Pittsburgh

## Abstract

To maintain core body temperature in mammals, the CNS thermoregulatory networks respond to cold exposure by increasing brown adipose tissue and shivering thermogenesis. However, in hibernation or torpor, this normal thermoregulatory response is supplanted by “thermoregulatory inversion”, an altered homeostatic state in which cold exposure causes inhibition of thermogenesis and warm exposure stimulates thermogenesis. Here we demonstrate the existence of a novel, dynorphinergic thermoregulatory reflex pathway between the dorsolateral parabrachial nucleus and the dorsomedial hypothalamus that bypasses the normal thermoregulatory integrator in the hypothalamic preoptic area to play a critical role in mediating the inhibition of thermogenesis during thermoregulatory inversion. Our results indicate the existence of a neural circuit mechanism for thermoregulatory inversion within the CNS thermoregulatory pathways and support the potential for inducing a homeostatically-regulated, therapeutic hypothermia in non-hibernating species, including humans.

## Introduction

The core body temperature (T_CORE_) of mammals is normally maintained within a narrow range that is optimal for enzymatic reactions and cellular function. The neural circuitry for normal thermoregulation alters the neural outflows to thermoeffector tissues in response to changes in skin and core temperatures to minimize deviations in T_CORE_^1^. In particular, brown adipose tissue (BAT) and skeletal muscle (shivering) thermogenesis are increased during exposure to a low ambient temperature (T_AMB_) and inhibited in a warm T_AMB_^2–6^. However, when certain mammals enter torpor or hibernation^7–9^, their brain circuits for thermoregulation switch from a normal to an inverted state in which the response to a low T_AMB_ is an inhibition of thermogenesis, which induces hypothermia and a reduction in energy consumption.

We have recently discovered that the central activation of A1-adenosine receptors induces a torpor-like state in rats^10–12^, and that the hypothermia and hypometabolism that characterize this torpor-like state arises from the induction of a novel thermoregulatory state of thermogenesis that we have called thermoregulatory inversion (TI)^10, 13^. In the TI state, as in natural torpor/hibernation, the CNS control of thermogenesis is inverted, such that thermogenesis is inhibited in a cold T_AMB,_ and stimulated in response to a warm T_AMB_.

In normal thermoregulation^1^, cold and warm signals from the skin thermoreceptors are transmitted via the dorsal horn to the parabrachial nuclei (PBN). Cold-responsive neurons in the external lateral PBN (elPBN)^4, 14^ and warm-responsive neurons in the dorsolateral PBN (dlPBN)^3, 14^ relay thermosensory signals to the preoptic area of the hypothalamus (POA). Responding to these cutaneous thermal signals, POA outputs regulate the balance of inhibitory^1, 15^ and excitatory^1, 16, 17^ inputs to thermogenesis-promoting neurons in the dorsomedial hypothalamus (DMH), that in turn excite thermogenesis premotor neurons in the medullary rostral raphe pallidus (rRPa)^1^. During skin cooling, excitation of thermogenesis-promoting DMH neurons prevails and thermogenesis is augmented. Conversely, in a warm environment, net inhibition of these DMH neurons reduces the excitatory drive to thermogenesis premotor neurons in the rRPa, and thermogenesis is reduced. Clearly, the neural circuitry supporting normal thermoregulation cannot be controlling T_CORE_ in torpor/hibernation, since in these states skin cooling inhibits thermogenesis and T_CORE_ falls^7, 18^.

The central neural circuitry underlying the control of thermogenesis during the TI state has not been delineated. The current study demonstrates the existence of novel thermoregulatory reflex pathways between the PBN and DMH bypassing the POA to control thermogenesis and T_CORE_ in the TI state. This novel circuit could represent the neural basis for the cooling-evoked hypothermia and hypometabolic state observed during natural torpor/hibernation, and could facilitate the induction of the TI state and a sustained hypothermia^10, 13, 19^ in species that do not have an endogenous ability to enter torpor/hibernation. Implementation of pharmacological and physiological strategies to induce therapeutic hypothermia and the resulting hypometabolism would have extensive clinical applications for increasing survival and recovery of function following ischemic incidents (stroke, cardiac arrest, neonatal encephalopathy), as well as for reducing metabolic demands during extended space flights.

## Results

### Inhibition of POA induces the TI state

Descending projections from the POA, including those to the DMH and rRPa, mediate the cutaneous and core temperature-evoked changes in BAT thermogenesis during normal thermoregulatory reflex responses^3, 4, 13, 15, 16, 20–23^. Neurons in the POA play a critical role in the induction of torpor in mice^8, 24,25^. Transection of pathways between the POA and the DMH (pre-DMH transX) establishes the novel thermoregulatory state of TI, in which the normal BAT thermogenic responses to skin cooling and skin warming are inverted^13^. To test the hypothesis that POA neuronal activity is required for the induction of the TI state, we determined the BAT sympathetic nerve activity (SNA) and thermogenic responses to skin cooling and to skin warming after injecting muscimol into the POA to inhibit local neurons.

**Figure 1:**
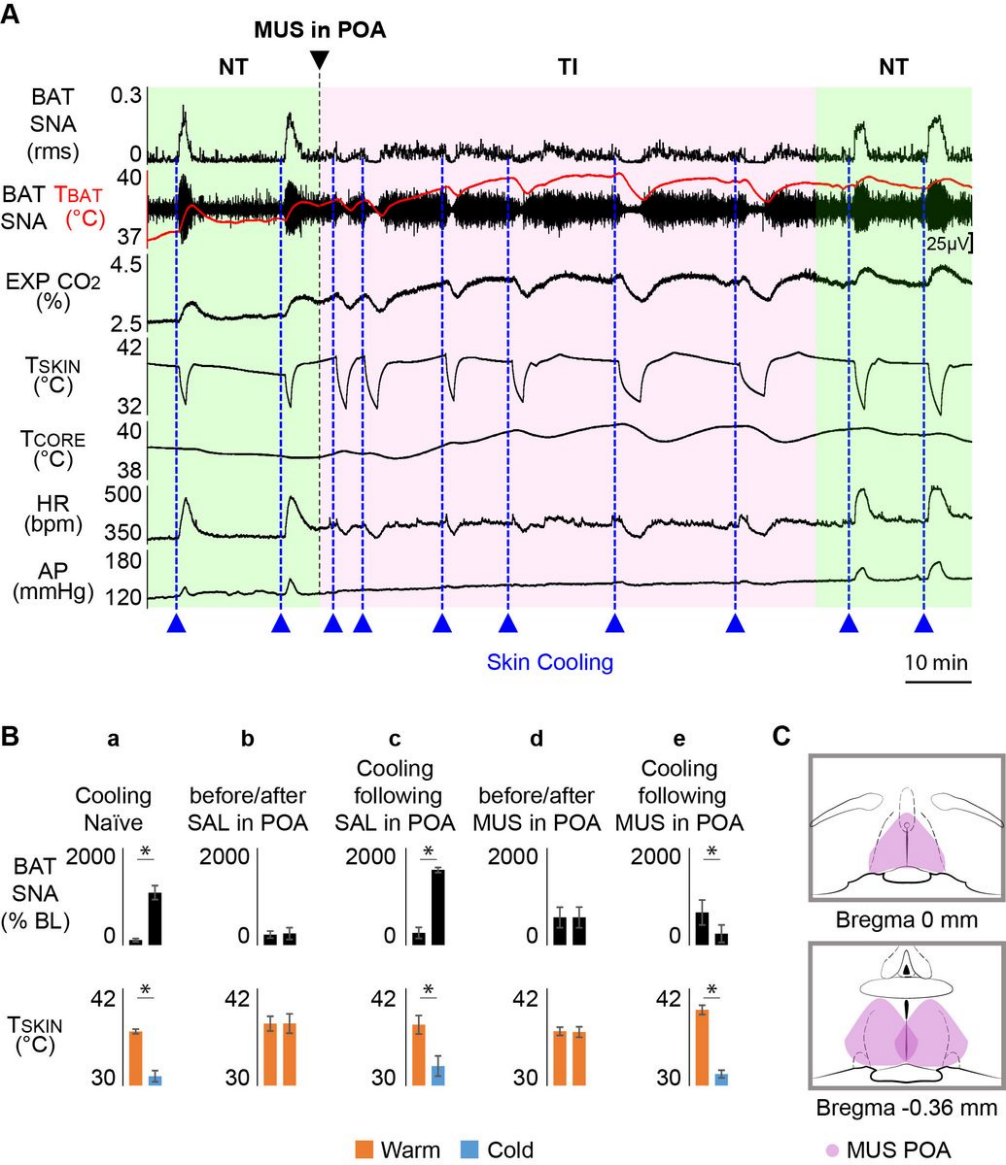
Inhibition of preoptic area (POA) neurons induces thermoregulatory inversion (TI) of brown adipose tissue (BAT) thermogenesis. **A**. During normal thermoregulation (NT) in an anesthetized, naïve rat, episodes of skin cooling (reductionsin skin temperature (T_SKIN_), dotted blue lines) produced an increase in BAT sympathetic nerve activity (SNA), BAT temperature (T_BAT_, BAT thermogenesis), expired CO_2_ (EXP CO_2_), heart rate (HR), and arterial pressure (AP). Skin rewarming inhibited BAT SNA and reversed all the effects of skin cooling. Bilateral nanoinjection of muscimol (MUS) into the POA did not alter the ongoing level of BAT SNA, but subsequent episodes of skin cooling elicited inhibitions of BAT SNA, and reductions in T_BAT_, EXP CO_2_, and HR. Skin rewarming reactivated BAT SNA and increased T_BAT_, EXP CO_2_, and HR. Such cooling-evoked inhibitions and warming-evoked activations of BAT SNA are the hallmark of the TI state. Normal thermoregulatory responses to skin cooling and skin rewarming returned as the pharmacological effect of MUS on POA neurons waned. **B.** Group data representing the changes in BAT SNA in response to changes in T_SKIN_. **a**. Skin cooling in naïve rats increased BAT SNA; **b**. the low BAT SNA during skin warming was unaffected by saline (SAL) injection in POA; **c**. skin cooling increased BAT SNA after SAL injection in POA; **d.** MUS injection in POA did not change the ongoing level of BAT SNA; **e**. after MUS injection in POA, BAT SNA is lower during skin cooling than during skin warming. *p < 0.05; T_CORE_: core temperature. **C.** Schematic of the observed extent and location of MUS nanoinjections in the POA.

In naïve, anesthetized rats, the normal thermoregulatory response to skin cooling is a prompt increase in BAT SNA (ΔT_SKIN_ = −5.6 ± 0.5°C from a baseline of 36.7 ± 0.3°C; ΔBAT SNA: + 983.9 ± 152.1% of precooling control; n = 10, p = 0.0001; Figs. 1A, 1Ba). Conversely, skin warming produces a strong inhibition of BAT SNA and thermogenesis (Fig. 1A). Bilateral nanoinjections of isotonic saline (vehicle) in the POA did not affect basal levels of BAT SNA (pre-saline in POA: 216.6 ± 74.9%BL; post-saline in POA: 238.8 ± 122.6%BL; n = 3, p = 0.7262, Fig. 1Bb), and skin cooling produced the stereotypic increase in BAT SNA (Δ T_SKIN_ = −5.1 ± 2.3°C; Δ BAT SNA: +1305.3 ± 166.6% of pre-saline control; n = 3, p = 0.0159; Fig. 1Bc) characteristic of a normal cold-defense response.

Bilateral nanoinjections of muscimol into the POA (Fig. 1C) did not alter the level of BAT SNA (ΔT_SKIN_ = −0.1 ± 0.2°C; Δ BAT SNA: +0.1 ± 83.7% of pre-muscimol control; n = 10, p = 0.4996; Figs. 1A, 1Bd). In marked contrast to saline nanoinjections in the POA (Fig. 1Bc), nanoinjections of muscimol into the POA induced the TI state in which skin cooling reduced BAT SNA (ΔT_SKIN_ = −8.9 ± 0.8°C from a baseline of 39.4 ± 0.6°C; Δ BAT SNA: 438.08 ± 103.0% of precooling control; n = 10, p = 0.0011; Figs. 1A, 1Be). Also characteristic of the TI state^13^, skin rewarming following muscimol injection in the POA increased BAT SNA and reversed the cooling-induced inhibition of BAT SNA (Figs. 1A, 1Be).

### Pre-dmh Transx Inverts The Normal Thermoregulatory Shivering Response

Since skeletal muscle shivering is the most signi cant source of thermoregulatory thermogenesis in humans^26^, it is important to determine if the thermoregulatory circuitry controlling shivering^22^ can also be manipulated to transition to the TI state in which cooling would inhibit shivering and allow T_CORE_ to fall as it does in torpor/hibernation.

### Partial pre-DMH transX eliminates cold-evoked shivering

**Figure 2:**
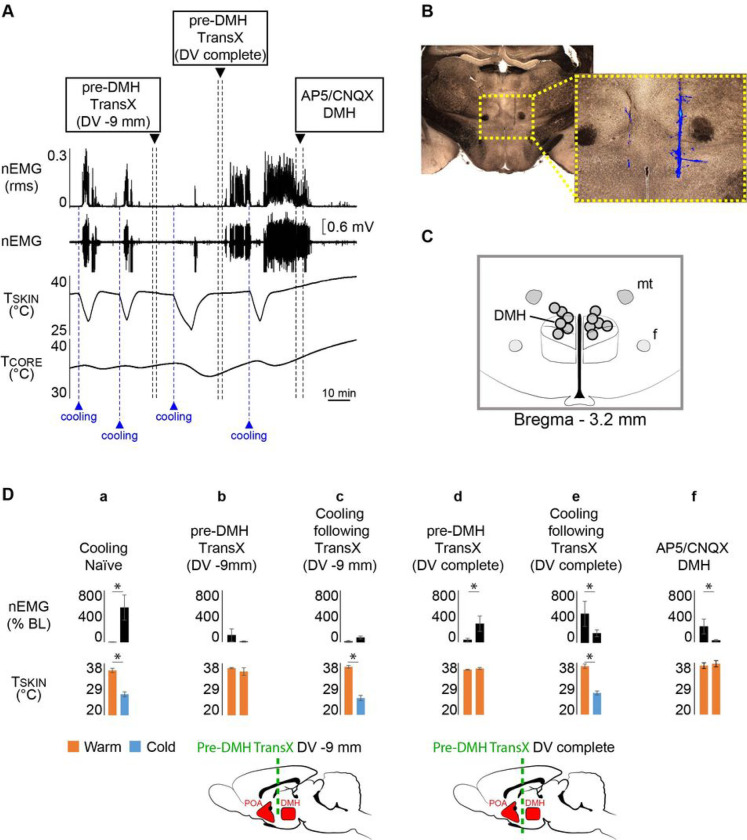
Pre-dorsomedial hypothalamus transection (Pre-DMH TransX) induces shivering thermoregulatory inversion (TI). **A**. During normal thermoregulation (NT) in an anesthetized, naïve rat, episodes of skin and core cooling (reductions in skin temperature (TSKIN), dotted blue lines) produced an increase in the neck muscle EMG (reductions in skin temperature (T_SKIN_), dotted blue lines) produced an increase in the neck muscle EMG (nEMG), indicative of shivering. Skin rewarming reversed the increases in nEMG. A pre-DMH transX to a depth of −9 mm ventral to brain surface (pre-DMH TransX, DV −9 mm) did not affect the low level of nEMG when T_CORE_ and T_SKIN_ were warm; however, skin and core cooling no longer activated nEMG. Completing the pre-DMH TransX to −10 mm produced an immediate increase in nEMG. Subsequent skin cooling inhibited nEMG, indicating the TI state. With the rat in the TI state, bilateral nanoinjection of AP5/CNQX into the DMH completely reversed the warm-evoked increases in nEMG. **B.** Histological section through the DMH illustrating the deposits of blue fluorescent beads indicating thelocation of bilateral nanoinjection sites of AP5/CNQX in DMH. **C.** Schematic of the observed location of AP5/CNQX nanoinjections into the DMH (n = 6). **D.** Group data representing the changes in nEMG (%BL: % baseline) in response to changes in T_SKIN_, pre-DMH TransX (schematic below bar graphs) (n = 9) or nanoinjection of AP5/CNQX in DMH (n = 6). **a.** Skin cooling in naïve rats increased nEMG; **b.** pre-DMH TransX to DV −9 mm did not alter the low level of nEMG in warm rats; **c**. after pre-DMH TransX to DV −9 mm, skin cooling no longer increased nEMG; **d.** complete pre-DMH TransX to −10 mm elicited an immediate increase in nEMG in warm rats; **e**. after complete pre-DMH TransX, nEMG was lower during skin cooling than during skin warming; f. in the TI state, after complete pre-DMH TransX, AP5/CNQX nanoinjections into the DMH eliminated the warm-evoked activation of nEMG. *p < 0.05; T_CORE_: core temperature; T_SKIN_: skin temperature.

In naïve, anesthetized rats, the normal thermoregulatory response to skin and core cooling is a prompt increase in shivering, registered as an increase in nuchal muscle EMG (nEMG) (ΔT_SKIN_ = −8.3 ± 1.1°C from a baseline of 35.56 ± 0.7°C; ΔnEMG: +526.6 ± 195.0% of pre-cooling control; n = 9, p = 0.002; Figs. 2A, 2Da). Conversely, skin rewarming produces a strong inhibition of nuchal muscle shivering (Fig. 2A), returning the nEMG to the low levels observed in warm rats.

A pre-DMH transX to −9 mm from the dorsal surface of the brain did not produce any change in nEMG in warm rats (T_SKIN_ = 36.4 ± 0.2°C; ΔnEMG: −99.9 ± 88.4% of pre-transX control; n = 5, p = 0.5; Figs. 2A, 2Db). However, as with BAT SNA^13^, this partial pre-DMH transX completely prevented the increase in shivering nEMG in response to skin cooling (ΔT_SKIN_ = −10.8 ± 1.2°C from a baseline of 36.7 ± 0.5°C; ΔnEMG: +57.1 ± 17.8% of pre-cooling control; n = 5, p = 0.125; Figs. 2A, 2Dc).

### Complete pre-DMH transX inverts the thermoregulatory shivering response

With T_CORE_ and T_SKIN_ in a warm condition and nEMG at a low, non-shivering level, a complete pre-DMH transX to −10 mm from the dorsal brain surface produced an immediate and remarkable increase in nEMG and nuchal muscle shivering (T_SKIN_ = 35.7 ± 0.1°C; ΔnEMG: +347.1 ± 145.7% of pre-transX control; n = 6, p = 0.0156; Figs. 2A, 2Dd). Paralleling the pre-DMH transX-evoked activation of BAT SNA in warm conditions^13^, the pre-DMH transX-induced activation of shivering nEMG in rats with a warm T_SKIN_ is indicative of the TI state. The TI state for shivering was con rmed by the demonstration that skin cooling inhibited these warming-evoked shivering nEMG responses. Following a pre-DMH transX, skin cooling consistently decreased nEMG (ΔT_SKIN_ = −10.3 ± 1.4°C from a baseline of 38.6 ± 0.9°C; ΔnEMG: −366.65 ± 212.3% of pre-cooling nEMG; n = 5, p = 0.0313; Figs. 2A, 2De). Subsequent skin rewarming consistently increased shivering nEMG (Fig. 2A).

### A glutamatergic excitation of DMH neurons is necessary for the skin warming-evoked increases in BAT SNA and BAT thermogenesis and in shivering EMG during TI

The CNS circuits for the normal cold-defensive activation of BAT and shivering thermogenesis require a glutamatergic activation of neurons in the DMH that project to thermogenic premotor neurons in the rRPa^1, 6, 16, 22, 27^. In the TI state, skin warming activates DMH neurons that project to rRPa (Extended Data Fig. 2). Is a glutamatergic excitation of thermogenesis-promoting neurons in the DMH also required for the skin warming-induced activation of BAT and shivering thermogenesis in the TI state (Figs. 1, 2)?

**Figure 3:**
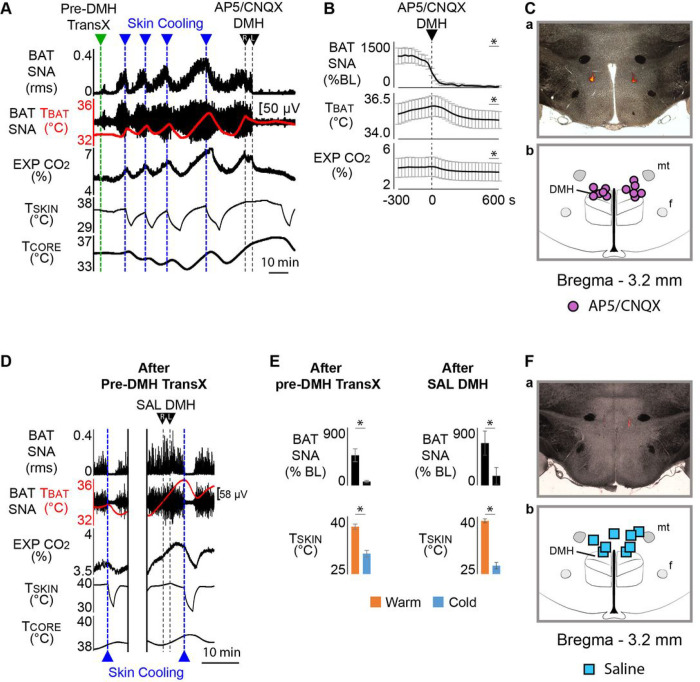
Following pre-DMH TransX, bilateral nanoinjection of AP5/CNQX in the DMH blocks the warm-evoked increase in BAT SNA. **A.** In the TI state following a complete pre-DMH TransX, episodes of skin cooling (reductions in skin temperature (T_SKIN_), dotted blue lines) produced the typical cold-evoked inhibition of BAT sympathetic nerve activity (SNA), and reductions in BAT temperature (T_BAT_, BAT thermogenesis) and expired CO_2_ (EXP CO_2_). Skin rewarming activated BAT SNA and reversed the effects of skin cooling. Bilateral nanoinjection of AP5/CNQX into the DMH produced a prompt and long-lasting inhibition of the warm-evoked activation of BAT SNA. **B.** Group data (n = 6) representing the changes in BAT SNA (% BL: % baseline), T_BAT_, and EXP CO_2_, in response to nanoinjection of AP5/CNQX into the DMH. *p < 0.05; T_CORE_: core temperature. **C. a.** An example of a histological section through the DMH illustrating the deposits of red fluorescent beads indicating the locations of the bilateral nanoinjection sites of AP5/CNQX in the DMH; **b.** schematic of the locations of the nanoinjection sites of AP5/CNQX in the DMH. **D.** Following a complete pre-DMH TransX, episodes of skin cooling (reductions in T_SKIN_, dotted blue lines) produced the cold-evoked inhibition of BAT SNA, T_BAT_, and EXP CO_2_, characteristic of the TI state. Bilateral nanoinjections of saline (SAL) into the DMH did not alter either the ongoing level of BAT SNA or the subsequent episodes of skin cooling-elicited inhibitions of BAT SNA and reductions in T_BAT_ and EXP CO_2_. **E.** Group data (n = 4) representing the changes in BAT SNA (% BL: % baseline), T_BAT_, and EXP CO_2_ in response to nanoinjections of SAL into the DMH. *p < 0.05. **F. a.** An example of a histological section through the DMH illustrating the deposits of red fluorescent beads indicating the locations of the bilateral nanoinjection sites of saline (SAL) in the DMH; **b.** schematic of the locations of the bilateral nanoinjections of SAL into the DMH.

Following a pre-DMH transX, the TI state was validated by demonstrating that skin warming (T_SKIN_ = 38.2 ± 0.6°C) resulted in an activation of BAT SNA (BAT SNA: 878.8 ± 184.3%BL; Fig. 3A). Subsequent bilateral nanoinjections of AP5/CNQX in the DMH (Fig. 3C) eliminated the warm-evoked increase in BAT SNA (ΔBAT SNA: −836.0 ± 194.4%BL; n = 6, p = 0.0039; Figs. 3A, 3B). The abrupt fall in BAT SNA resulted in a decrease in T_BAT_ (−0.7 ± 0.1°C, n = 6, p = 0.0018) and in expired CO_2_ (−0.4 ± 0.1%, n = 6, p = 0.0044; Fig. 3B).

In rats in the TI state after a pre-DMH transX and with a warm skin (T_SKIN_ = 38.5 ± 1.1°C) and an actively shivering nEMG (243.7 ± 114.2%BL; Fig. 2A), bilateral nanoinjections of AP5/CNQX in the DMH (Figs. 2B, 2C) reversed the warm-evoked activation of shivering nEMG (ΔnEMG: −217.0 ± 104.1%BL; n = 6, p = 0.0313; Figs. 2A, 2Df).

In the TI state after pre-DMH transX, bilateral nanoinjection of saline (vehicle) in the DMH (Fig. 3F) had no effect on the skin warming-evoked activation of BAT SNA (pre-saline BAT SNA: 750.2 ± 306.1%BL, post-saline BAT SNA: 700.9 ± 230.4%BL; n = 4, p = 0.3260; Figs. 3D, 3E). Additionally, the cold-evoked inhibition of BAT SNA characteristic of the TI state was unaffected by saline nanoinjections in the DMH (ΔT_SKIN_ = −11.7 ± 0.8°C from a baseline of 38.9 ± 0.5°C; ΔBAT SNA: −513.2 ± 108.3% of pre-cooling control; n = 4, p = 0.0089; Figs. 3D, 3E).

These results indicate that in the TI state increases in BAT and shivering thermogenesis are dependent on a glutamatergic excitation of thermogenesis-promoting neurons in the DMH. Since a complete pre-DMH transX (Fig. 2D) or a nanoinjection of muscimol in POA (Fig. 1C) induces a robust TI state, it seems unlikely that the source of the glutamatergic input to the DMH required for the skin warming-evoked activation of BAT and shivering thermogenesis in the TI state is located within the POA, as it is for normal thermoregulation ^16^, but rather from neurons located caudal to the pre-DMH transX.

To provide evidence that in the TI state skin warming activates DMH neurons that project to rRPa, we examined the Fos expression (Fos-ir) in DMH neurons that were retrogradely-labeled with FluoroGold (FG) injected into the rRPa in warm-exposed rats after a pre-DMH transX (Warm-T rats). A significantly higher percentage of rRPa-projecting (FG-ir) neurons in the DMH were double-labeled (FGFos) in Warm-T rats than in Cold-T rats (Warm-T: 22.38 ± 4.01% FGFos/FG vs. Cold-T: 11.34 ± 1.3% FGFos/FG, n = 4, p = 0.02319, Extended Data Fig. 2).

### A glutamatergic excitation of PBN neurons is necessary for the skin warming-evoked increase in BAT SNA during TI

The central afferent pathways from cutaneous thermoreceptors, which drive a glutamatergic excitation of neurons in two subnuclei of the PBN (dlPBN and elPBN^3, 4, 14^), comprise the sensory components for the normal thermoregulatory reflex control of thermogenesis. Is a glutamatergic excitation of PBN neurons also essential for the inverted skin thermoreceptor regulation of thermogenesis in the TI state?

**Figure 4:**
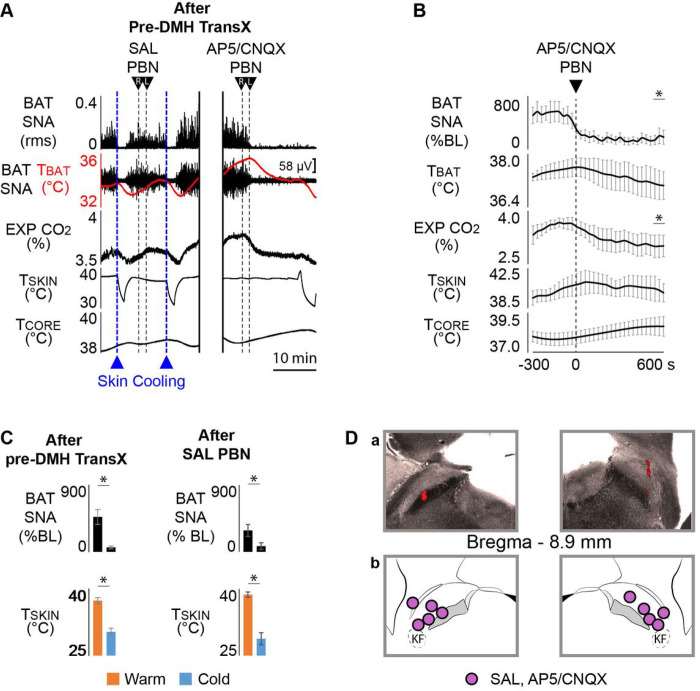
Bilateral nanoinjections of AP5/CNQX in the parabrachial nuclei (PBN) blocked the warm-evoked BAT SNA characteristic of TI. **A.** In the TI state following a complete pre-DMH TransX, episodes of skin cooling (reductions in T_SKIN_ dotted blue lines) produced the typical cold-evoked inhibition of BAT SNA and reductions in T_BAT_ and expired CO_2_ (EXP CO_2_). Skin rewarming activated BAT SNA and reversed the effects of skin cooling. Bilateral nanoinjections of saline (SAL) into the PBN did not alter either the ongoing level of BAT SNA or the inhibitions of BAT SNA and reductions in T_BAT_ and EXP CO_2_ during skin cooling. Subsequent nanoinjections of AP5/CNQX into the PBN produced a prompt and long-lasting inhibition of the warm-evoked activation of BAT SNA and reduced T_BAT_ and EXP CO_2_. **B.** Group data (n = 5) representing the time course of the reductions in BAT SNA (% BL: % baseline) andEXP CO_2_ in response to nanoinjections of AP5/CNQX into the PBN. *p < 0.05; T_CORE_: core temperature; T_BAT_: BAT temperature. **C.** Group data (n = 5) representing the levels of BAT SNA (% BL: % baseline) vs. T_SKIN_. Following a complete pre-DMH TransX, BAT SNA was elevated during skin warming, typical of the TI state. BAT SNA remained higher during skin warming than during skin cooling after nanoinjections of SAL into PBN. *p < 0.05. **D. a.** An example of a histological section through the PBN illustrating the deposits of red fluorescent beads indicating the locations of the bilateral nanoinjection sites of SAL and of AP5/CNQX in the PBN; **b.** schematic of the locations of the bilateral nanoinjections of SAL and of AP5/CNQX into the PBN.

In the TI state following a pre-DMH transX, skin cooling consistently decreased BAT SNA (ΔT_SKIN_ = −7.0 ± 1.1°C from a baseline of 37.4 ± 0.7°C; ΔBAT SNA: −411.4 ± 98.4% of pre-cooling control; n = 5, p = 0.0007; Figs. 4A, 4C) and skin rewarming consistently increased BAT SNA (ΔT_SKIN_ = + 6.6 ± 1.3°C from a baseline of 30.4 ± 0.8°C; ΔBAT SNA: +359.1 ± 89.3% of pre-cooling control; n = 5, p = 0.0012; Figs. 4A, 4C). During skin warming (T_SKIN_ = 40.6 ± 1.0°C, n = 5) and an elevated BAT SNA (515.11 ± 130.7%BL; n = 5), bilateral nanoinjections of AP5/CNQX into the PBN (Fig. 4D) promptly decreased BAT SNA (ΔBAT SNA: −329.6 ± 112.5%BL, n = 5, p = 0.0313; Figs. 4A, 4B). The long-lasting (> 1 hr) inhibition of the skin warming-evoked activation of BAT SNA resulted in a decrease in T_BAT_ (−0.6 ± 0.2°C, n = 3, p = 0.0355) and in expired CO_2_ (−0.2 ± 0.1%, n = 5, p = 0.0387; Fig. 4B). Thus, in the TI state, the inverted control of thermogenesis by skin thermoreceptors requires a glutamatergic activation of neurons in the PBN.

In the TI state and with the skin kept warm, bilateral nanoinjections of isotonic saline (vehicle) in the PBN had no effect on the warm-evoked activation of BAT SNA (pre-saline in PBN BAT SNA: 333.5 ± 134.4%BL, post-saline in PBN BAT SNA: 281.33 ± 80.5%BL; n = 5, p = 0.5231; Figs. 4A, 4C). In the TI state, nanoinjection of saline in the PBN also had no effect on the characteristic cold-evoked inhibition of BAT SNA (ΔT_SKIN_ = −9.9 ± 1.8°C from a baseline of 38.7 ± 0.6°C; ΔBAT SNA: −212.2 ± 52.6% of pre-cooling BAT SNA; n = 4, p = 0.0089; Figs. 4A, 4C).

### PBN neurons projecting to DMH are activated during the inverted thermoregulatory thermogenic responses in the TI state

The skin thermoreceptor-mediated modulation of thermogenesis in the TI state requires both the descending thermogenesis-promoting pathways from DMH to the rRPa (Figs. 2,3; Extended Data Fig. 2), as well as an ionotropic glutamate receptor-mediated excitation of neurons in the specific regions of the PBN (Fig. 4) receiving thermosensory signals from second-order thermosensory neurons in the spinal dorsal horn. Since the inverted regulation of thermogenesis in the TI state occurs in the absence of direct POA inputs to the DMH (Figs. 1, 2, 3), we tested the hypothesis that PBN neurons with direct projections to the DMH are activated during skin thermoreceptor stimulation in the TI state.

DMH-projecting neurons in the PBN are activated during skin warming in anesthetized, naïve rats and in anesthetized, pre-DMH transX rats.

**Figure 5:**
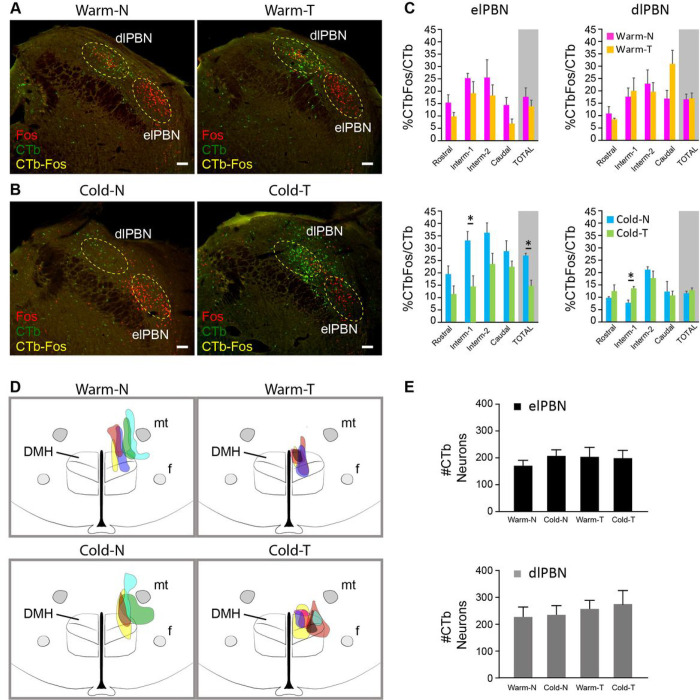
Effect of warm and cold exposure on the activation of DMH-projecting neurons in the PBNfollowing pre-DMH TransX. **A.** An example of the distribution of neurons double-labeled for warm-evoked Fos (red nucleus) and for CTb retrogradely transported from DMH (green cytoplasm) in the PBN of anesthetized naïve rats (Warm-N) and anesthetized pre-DMH TransX rats (Warm-T). The dotted yellow circles in dlPBN and elPBN represent the counting boxes used for the quantitative analysis reported in panel C. **B.** An example of the distribution of neurons double-labeled for warm-evoked Fos (red nucleus) andretrogradely transported CTb from DMH (green cytoplasm) in the parabrachial nuclei (PBN) of anesthetized naïve rats (Cold-N) and anesthetized pre-DMH TransX rats (Cold-T). The dotted yellow circles in dlPBN and in elPBN represent the counting boxes used for the quantitative analysis reported in panel C. **C.** Group data representing the changes in the percentage of retrogradely-labeled neurons (% CTbFos/CTb) in dlPBN and elPBN at the different rostrocaudal levels of PBN that also exhibited Fos in response to the four different treatments: Warm-N, Warm-T, Cold-N, and Cold-T. *p < 0.05. **D.** Schematic of the location and diffusion of unilateral (right) nanoinjection of the retrograde tracer, cholera toxin subunit B (CTb), into the DMH of the four treatment groups: Warm-N, Warm-T, Cold-N, and Cold-T. **E.** Group data indicating the counts (#CTb) of dlPBN and elPBN neurons retrogradely-labeled from CTbinjections in the DMH in the different treatment groups: Warm-N, Warm-T, Cold-N, and Cold-T. There were no differences in CTb counts among the 4 groups. Scale bar in all images = 100 μm.

We compared anatomical assessments of PBN neuronal activation (Fos-ir) in 4 groups of anesthetized rats: naïve rats and pre-DMH transX rats during skin warming and during skin cooling. Our injections of the retrograde tracer, cholera toxin subunit b (CTb), in the DMH overlapped with DMH neurons retrogradely-labeled following injections of another retrograde tracer, Fluorogold (FG), in the rRPa (Extended Data Figs. 1A, 1B), and resulted in CTb retrograde labeling of neurons in the elPBN and dlPBN (Extended Data Fig. 1C). This basic anatomical result is consistent with the potential for PBN neurons to directly influence the activity of thermogenesis-promoting neurons in the DMH. There was no difference between the number of CTb-ir neurons in the elPBN and in the dlPBN in the 4 treatment groups (p > 0.05, Fig. 5E). To analyze the extent of Fos expression in dlPBN and elPBN neurons (Fig. 5A) that were retrogradely labeled from CTb injections in DMH (Fig. 5D), we calculated the percent of CTb-labeled neurons in elPBN and in dlPBN that were also Fos-ir (% CTbFos/CTb; Fig. 5C) in PBN sections at 4 consecutive rostro-caudal levels separated by approximately 100 μm.

During skin warming in anesthetized, naive rats (Warm-N), we observed Fos-ir in both dlPBN (total: 16.60 ± 2.06%) and elPBN (total: 17.75 ± 3.58%) neurons that projected to DMH (Figs. 5A, 5C). In anesthetized pre-DMH transX rats with a warm skin (Warm-T), we also found Fos-ir DMH-projecting neurons in both dlPBN (total: 16.93 ± 2.19%) and elPBN (total: 13.89 ± 2.50%) (Figs. 5A, 5C), and at comparable levels to those observed in the Warm-N rats.

Pattern of activation of DMH-projecting neurons in the PBN in anesthetized, naive rats and in anesthetized, pre-DMH transX rats after cold exposure

Skin cooling in anesthetized, naïve rats (Cold-N), a condition in which thermogenesis is strongly stimulated, activated 27 ± 0.95% of the total elPBN neurons that project to DMH (Figs. 5B, 5C). Skin cooling in anesthetized, pre-DMH transX rats (Cold-T), a condition in which thermogenesis is inhibited, activated significantly fewer DMH-projecting neurons in elPBN (14.73 ± 2.31%; n = 5, p = 0.003; Figs. 5B, 5C) than in the Cold-N group. This reduction was most prominent at the Intermediate-1 level of the elPBN (Cold-N: 33.09 ± 3.65% vs. Cold-T: 14.52 ± 4.27%, n = 5, p = 0.0151; Fig. 5C). Skin cooling in anesthetized, naive rats (Cold-N) also activated DMH-projecting neurons in dlPBN (11.79 ± 0.81%). A similar fraction (12.98 ± 0.91%) of the DMH-projecting neurons in dlPBN was also activated by skin cooling in anesthetized, pre-DMH transX rats (Cold-T) (Figs. 5B, 5C). These data support the idea that in anesthetized, naive rats, activation of DMH-projecting neurons in elPBN and in dlPBN contributes to the regulation of the discharge of thermogenesis-promoting neurons in DMH during normal thermoregulatory cold defense. In addition, our nding that the activation of DMH-projecting neurons in elPBN was lower in Cold-T than in Cold-N rats would be consistent with a reduced excitation of DMH neurons from their elPBN inputs in the TI state, when skin cooling reduces thermogenesis (Figs. 1, 2, 3).

PBN neurons with direct projections to the DMH are activated during warm and cold exposure in naïve, free-behaving rats

We sought to establish that PBN neurons with projections to the DMH were also activated during skin thermoreceptor stimulation in free-behaving rats. One week following CTb injections in the DMH, these naïve, free-behaving rats were exposed to either a warm or a cold T_AMB_, and Fos expression was quanti ed in DMH-projecting PBN neurons. Since there was no difference between the number of elPBN and dlPBN CTb-retrogradely neurons in warm- or cold-exposed free-behaving rats (elPBN: 140.0 ± 12.93 vs. 175.8 ± 7.56, n = 5, p = 0.06; dlPBN: 114.0 ± 10.63 vs. 146.6 ± 22.06, n = 5, p = 0.2275), we expressed the double-labeled PBN neuron counts as a percentage of the number of retrogradely-labeled PBN neurons (% CTbFos/CTb) throughout the dlPBN and elPBN subdivisions of the PBN.

**Figure 6:**
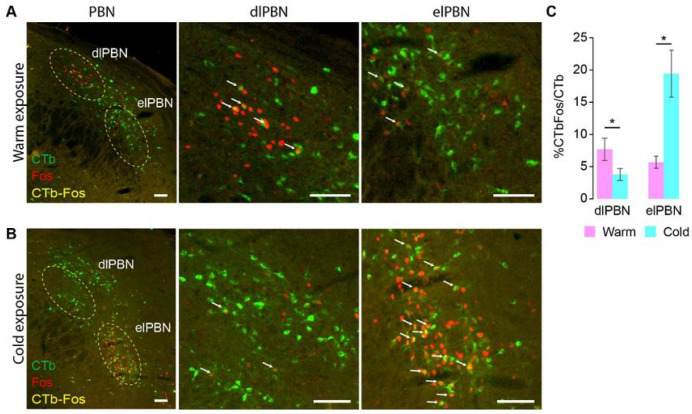
Anatomical identification of the DMH-projecting neurons in PBN activated during warm and cold exposure in free-behaving, naïve rats. **A.** Left picture: an example of the distribution of neurons double-labeled for warm-evoked Fos (red nucleus) and retrogradely transported CTb from DMH (green cytoplasm) in the parabrachial nuclei (PBN). The dashed yellow circles in the dorsolateral PBN (dlPBN) and in the externolateral PBN (elPBN) represent the counting boxes used for the quantitative analysis reported in panel C. The middle and right pictures show the dlPBN and elPBN, respectively, at higher magni cation. White arrows indicate a few examples of warm-activated neurons projecting to DMH (CTbFos) in dlPBN and elPBN. **B.** Left picture: an example of the distribution of neurons double-labeled for cold-evoked Fos (red nucleus) and retrogradely transported CTb from DMH (green cytoplasm) in the PBN. The dashed yellow circles in dlPBN and elPBN represent the counting boxes used for the quantitative analysis reported in panel C. The middle and right pictures show the dlPBN and elPBN, respectively, at higher magni cation. White arrows indicate a few examples of cold-activated neurons projecting to DMH (CTbFos) from the dlPBN and the elPBN. **C.** Group data representing the changes in the percentage of retrogradely-labeled neurons (% CTbFos/CTb) in dlPBN and elPBN responding to warm (pink) and cold exposure (blue). *p < 0.05. Scale bar in all images =100 μm.

During warm exposure in naïve, free-behaving rats, a condition in which we expect low levels of thermogenesis, we observed similar percentages of CTbFos/CTb in DMH-projecting neurons in the dlPBN (7.68 ± 1.74%) and within elPBN (5.67 ± 0.95%) (Figs. 6A, 6C). During cold exposure, when thermogenesis should be activated, Fos-ir was also observed in DMH-projecting neurons within dlPBN (3.78 ± 0.93%) and within elPBN (19.41 ± 3.64%) (Figs. 6B, 6C). Of the dlPBN neurons that projected to DMH, a significantly greater percentage expressed Fos-ir in warm-exposed rats than in cold-exposed rats (n = 5 per group; p = 0.0416; Fig. 6C). In contrast, for elPBN neurons that projected to the DMH, a significantly greater percentage expressed Fos-ir in cold-exposed rats than in warm-exposed rats (n = 5 per group; p = 0.0032; Fig. 6C). In these same rats, we observed FG-ir neurons in the DMH that expressed Fos-ir after cold exposure (Extended Data Fig. 2A). These anatomical data indicate that neurons in both the dlPBN and the elPBN project to the region of the DMH containing thermogenesis-promoting neurons (Fig. S1C). These populations of PBN neurons take part in the control of DMH thermoregulatory neurons during normal thermoregulation, in naïve, free-behaving rats.

### Dynorphinergic Neurons In Pbn Project To Dmh

The region of the PBN containing DMH-projecting neurons (Figs. 5A, 6A, 6B) also contains Dynorphin (Dyn) neurons^14, 28^. The intermediate-1 level subpopulation of DMH-projecting neurons in dlPBN is activated during skin cooling in pre-DMH transX rats (Fig. 5C), a TI state in which skin cooling inhibits thermogenesis by reducing the discharge of DMH neurons. These ndings, coupled with Dyn exerting an inhibitory influence on neuronal activation (through activation of κ-opioid receptors^29–31^), prompted us to test the hypothesis that Dyn, potentially released from terminals of the PBN neurons projecting to the DMH, plays a role in the cooling-evoked inhibition of thermogenesis characteristic of the TI state (Figs. 1, 2). Initially, we sought to determine if any of the Dyn neurons in the PBN project to the region of the DMH containing thermogenesis-promoting neurons, and if such a neuronal population is activated during normal thermoregulation and/or in the TI state.

**Figure 7:**
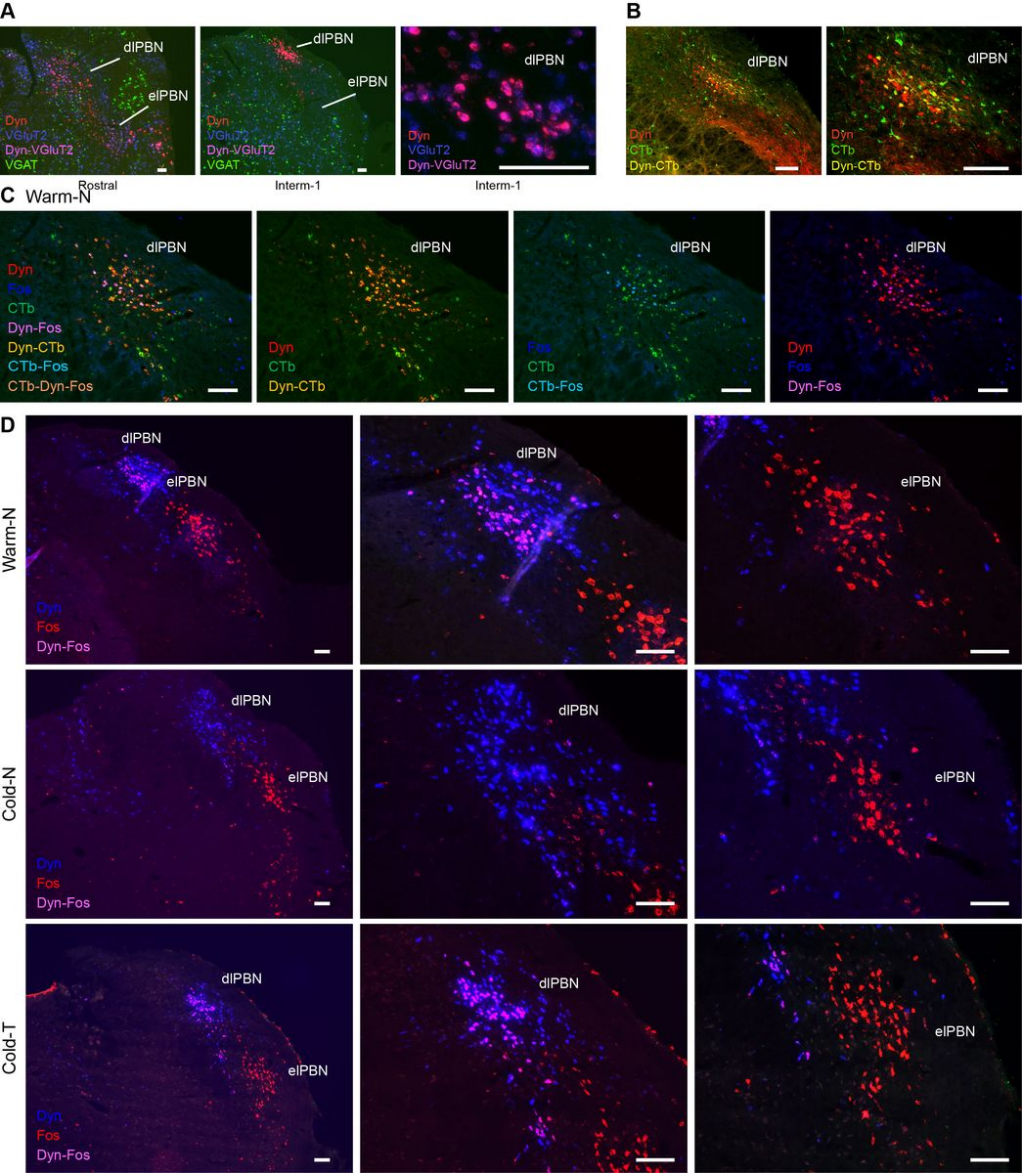
VGluT2-expressing Dynorphinergic neurons in PBN project to DMH and are activated during TI. **A.** Dynorphinergic neurons (containing pDyn transcripts; red) were observed in several PBN subdivisions along its rostro-caudal extent, but the strongest labeling was in dense clusters located in the dlPBN at the intermediate-1 and -2 levels. All Dyn neurons in these clusters express VGluT2 transcripts (blue), but none expresses VGAT transcripts (green). Neurons expressing both pDyn and VGluT2 transcripts display pink fluorescence. Samples were processed for ISH with RNAScope. **B.** A subset of DMH-projecting neurons in the dlPBN (green), retrogradely labeled with CTb, colocalize Dyn(red) in rats treated with colchicine to concentrate the neuropeptide in the cell bodies. Double-labeled neurons (with IHC) display yellow fluorescence. **C.** Example of DMH-projecting (CTb labelingwith IHC; green) Dyn (pDyn transcripts with ISH; red) neuronsin the dlPBN activated (c-fos transcripts with ISH; blue) during skin warming in an anesthetized naïve rat (Warm-N), a condition in which we expect thermogenesis to be inhibited. Triple-labeled neurons (CTb-pDyn-c-fos) display light brown fluorescence. Double-labeled neurons CTb-pDyn appear yellow, double-labeled neurons CTb-c-fos appear cyan, whereas double-labeled neurons pDyn-c-fos appear pink. **D.** Fewer Dyn neurons (pDyn transcripts with ISH; blue) in the dlPBN were activated (c-fos transcripts withISH; red) in anesthetized naïve rats exposed to skin cooling (Cold-N) than in either anesthetized naïve rats during skin warming (Warm-N) or pre-DMH TransX rats subjected to skin cooling (Cold-T). These observations are consistent with a thermogenesis-inhibiting role for these DMH-projecting Dyn neurons in the dlPBN, also supported by our nding that more of them are activated in Warm-N and Cold-T rats when thermogenesis is inhibited than in Cold-N rats, when thermogenesis is active. Scale bar in all images = 100 μm.

To identify Dyn neurons in the PBN and whether they express vesicular glutamate transporter 2 (VGluT2) or vesicular GABA transporter (VGAT), we performed in situ hybridization (ISH) with RNAScope on sequential brain sections containing the PBN. Dyn neurons were observed in several PBN subdivisions along its entire rostro-caudal extent (Fig. 7A), but the strongest transcript labeling was in dense clusters located in the dlPBN at the intermediate-1 and − 2 levels (Fig. 7A). All Dyn neurons in these clusters express VGluT2 transcripts (in a sample of 375 Dyn neurons in 2 sections counted bilaterally, all neurons colocalized VGluT2). None of the Dyn neurons in PBN express VGAT transcripts (Fig. 7A).

To determine if the Dyn neurons in the PBN project to the DMH, we performed immunohistochemistry (IHC) for Dyn and CTb in brains from rats that had been injected with CTb in DMH and treated with intracerebroventricular (ICV) colchicine. We observed colocalization of CTb and Dyn mainly in the dense clusters of Dyn neurons in the dlPBN (Fig. 7B). Thus, there is a concentration of VGluT2-expressing Dyn neurons in the dlPBN region, and many of these Dyn neurons project to the region of the DMH that contains thermogenesis-promoting neurons.

To determine whether the activity of DMH-projecting Dyn neurons in dlPBN could influence the level of thermogenesis in normal thermoregulation or in the TI state, we performed a co-detection procedure to identify DMH-projecting (CTb labeling with IHC) Dyn (pDyn transcripts with ISH) neurons in PBN that were activated (c-fos with ISH) by cutaneous thermal stimuli in naïve and pre-DMH transX rats. We observed DMH-projecting (CTb) Dyn neurons in dlPBN that were activated (c-fos) during skin warming in anesthetized naïve (Warm-N) rats (Fig. 7C), a condition in which we expect thermogenesis to be inhibited. Noticeably, fewer Dyn neurons in the dlPBN were activated in Cold-N rats than in either Warm-N or Cold-T rats (Fig. 7D). Our observation that DMH-projecting Dyn neurons in the dlPBN are activated in Warm-N rats, when inhibitory influences on the discharge of thermogenesis-promoting neurons in DMH predominate, is consistent with a signi cant thermogenesis-inhibiting role for these DMH-projecting Dyn neurons in the dlPBN. Such a role for Dyn neurons in the dlPBN is also supported by our nding that more of them are activated in Warm-N and Cold-T rats when thermogenesis is inhibited than in Cold-N rats, when thermogenesis is active (Fig. 7D).

### Dynorphin In Dmh Inhibits Normal, Cold-evoked Bat Sna And Bat Thermogenesis

Having identified a dynorphinergic projection from the PBN to the region of the DMH containing thermogenesis-promoting neurons, we sought to determine if Dyn in the DMH would affect normal, cold-evoked BAT SNA and BAT thermogenesis. Since the degradation products of exogenous Dyn by extracellular peptidases lead to non-specific activation of NMDA receptors^32^, we pretreated the DMH with the peptidase inhibitor Amastatin.

**Figure 8:**
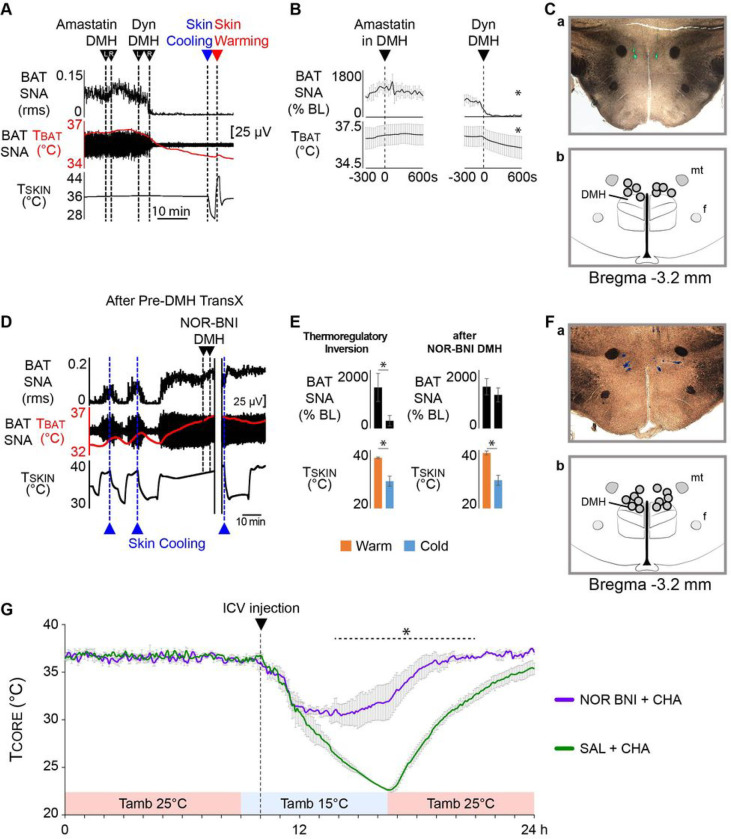
Activation of κ-opioid receptors is necessary for the cold-evoked inhibition of BAT SNA in the TI and torpor-like state. **A.** During normal thermoregulation (NT) in an anesthetized, naïve rat, skin cooling (reductions in skin temperature (T_SKIN_)) was used to elicit a sustained increase in BAT sympathetic nerve activity (SNA) and BAT temperature (T_BAT_, BAT thermogenesis). Bilateral nanoinjections of dynorphin (Dyn) into the DMH produced a prompt and complete inhibition of the cooling-evoked level of BAT SNA and resulted in a strong decrease of T_BAT_. Bilateral injection of the protease inhibitor, Amastatin, into DMH was used to inhibit Dyn cleavage. **B.** Group data (n = 4) representing the time course of the changes in BAT SNA (% BL: % baseline), and in T_BAT_ following nanoinjections of either Amastatin or Dyn into DMH. *p < 0.05; T_BAT_: BAT temperature. **C. a**. An example of a histological section through the DMH illustrating the deposits of green fluorescent beads indicating the locations of the bilateral nanoinjection sites of Amastatin and Dyn into the DMH; **b.** schematic of the locations of the bilateral nanoinjections of Amastatin and Dyn into the DMH. **D.** In the TI state following a complete pre-DMH TransX, episodes of skin cooling (reductions in T_SKIN_, dotted blue lines) produced the typical cold-evoked inhibition of BAT SNA. Skin rewarming activated BAT SNA and reversed the effects of skin cooling. Bilateral nanoinjections of the κ-opioid antagonist, NOR-BNI, into the DMH completely prevented the cold-evoked inhibition of BAT SNA. **E.** Group data (n = 6) representing the levels of BAT SNA (% BL: % baseline) vs. T_SKIN_. Following a complete pre-DMH TransX, BAT SNA was elevated during skin warming and reduced by skin cooling. Nanoinjection of NOR-BNI into the PBN eliminated the skin cooling-evoked decrease in BAT SNA, which remained elevated during skin cooling to a level comparable to that during skin warming. *p < 0.05. **F. a.** An example of a histological section through the DMH illustrating the deposits of blue fluorescent beads indicating the locations of the bilateral nanoinjections sites of NOR-BNI into DMH; **b.** schematic of the locations of the bilateral nanoinjections of NOR-BNI into DMH. **G.** Group data (n = 4) representing the mean ± SEM time course of the reductions in core temperature (T_CORE_) in response to ICV injection of either NOR BNI + CHA (purple trace) or SAL + CHA (green trace) in free-behaving rats maintained in a cold ambient temperature (T_AMB_). *p < 0.05.

In anesthetized naïve rats with a cool skin (T_SKIN_ = 35.2 ± 0.4°C) and an activated BAT SNA, bilateral nanoinjections of Amastatin into the DMH (Fig. 8C) did not affect BAT SNA (pre-Amastatin: 787.5 ± 33.0% BL, post-Amastatin: 816.7 ± 163.06% BL, n = 4, p = 0.8459; Figs. 8A, 8B) or T_BAT_ (pre-Amastatin: 36.4 ± 0.7°C, post-Amastatin: 36.6 ± 0.6°C; n = 4, p = 0.0546; Figs. 8A, 8B). Subsequent nanoinjections of Dyn in the same site promptly inhibited normal, cold-defensive BAT SNA (pre-Dyn: 613.0 ± 116.6%BL, post-Dyn: 61.7 ± 39.4%BL; n = 4, p = 0.0142), which caused a signi cant decrease in T_BAT_ (pre-Dyn: 36.2 ± 0.6°C, post-Dyn: 36.8 ± 0.7°C; n = 4, p = 0.0469; Figs. 8A, 8B). Following Dyn nanoinjection in the DMH, subsequent skin cooling no longer activated BAT SNA (Fig. 8A). Additionally, skin warming had no effect on the post-Dyn completely inhibited level of BAT SNA (Fig. 8A), indicating that Dyn nanoinjection in the DMH does not induce the TI state in which skin warming activates BAT SNA (cf. Figures 1, 3).

### A κ-opioid receptor antagonist in the DMH prevents the cold-evoked inhibition of BAT thermogenesis during TI

Since a population of Dyn-expressing neurons in PBN projects to the DMH, and Dyn nanoinjection into the DMH inhibits normal, cold-evoked BAT SNA and reduces BAT thermogenesis, we tested the hypothesis that Dyn, acting via k-opioid^33^ receptors in the DMH, contributes to the cold-evoked inhibition of BAT SNA in the TI state.

In the TI state following pre-DMH transX, skin cooling consistently decreased BAT SNA (ΔT_SKIN_ = −8.0 ± 1.5°C from a baseline of 37.3 ± 0.3°C; ΔBAT SNA: −1139.9 ± 323.9%BL; n = 6, p = 0.0085; Figs. 8D, 8E). With a warm T_SKIN_ (39.0 ± 0.7°C) and an active BAT SNA (1426.9 ± 277.0%BL), bilateral nanoinjections of nor-BNI in the DMH (Fig. 8F) prevented any subsequent cold-evoked inhibitions of BAT SNA (ΔT_SKIN_ = −9.3 ± 2.2°C from a baseline of 39.0 ± 0.7°C; ΔBAT SNA: −280.4 ± 172.6%BL; n = 6, p = 0.0825; Fig. 8D, 8E). Thus, in the TI state, nor-BNI administration into the DMH resulted in an 82.1 ± 19.2% reduction in the cold-evoked inhibition of BAT SNA, indicating that Dyn release in the DMH is necessary for the skin cooling-evoked reduction in thermogenesis in the TI state.

### Blockade of central κ-opioid receptors reduces the hypothermic response to ICV administration of an adenosine 1A receptor agonist in free-behaving rats

In free-behaving rats exposed to a cool T_AMB_, central administration of the adenosine 1A receptor (A1A-R) agonist, CHA, produces a progressive hypothermia consistent with an inverted regulation of thermogenesis, characteristic of the TI state^13^. Since Dyn release in the DMH is required for the skin cooling-evoked inhibition of thermogenesis in the TI state in anesthetized rats (Figs. 8D, 8E), we determined if blockade of central κ-opioid receptors with ICV nor-BNI would affect the cooling-evoked hypothermia during the TI state induced by the central administration of CHA in free-behaving rats.

One hour after reducing the T_AMB_ from 25°C to 15°C, free-behaving rats chronically instrumented for T_CORE_ recording, received an ICV pretreatment of either 0.9% saline vehicle (5 μl) or nor-BNI, followed after 10 minutes by an ICV injection of CHA (1 mM, 5μl). During the 1 h exposure to the T_AMB_ of 15°C prior to pretreatment, the rats maintained a normal T_CORE_ of 36.7 ± 0.1°C (n = 3; Fig. 8G), re ecting a normal thermoregulatory cold-defense response. Following saline pretreatment, administration of CHA elicited a prompt reduction in T_CORE_ (Fig. 8G), which reached a minimum of 22.4 ± 0.2°C (ΔT_CORE_ = −14.2 ± 0.2°C from a baseline of 36.7 ± 0.2°C, n = 3) at 7 h:36 min ± 7 min following CHA injection (Fig. 8G). Following pretreatment with nor-BNI, injection of CHA also elicited a rapid reduction in T_CORE_ (Fig. 8G). However, the fall in T_CORE_ (ΔT_CORE_ = −7.2 ± 0.8°C from a baseline of 36.7 ± 0.3°C, n = 3) following CHA administration was significantly less after pretreatment with nor-BNI than after saline pretreatment (p < 0.001, Bonferroni post-hoc test). Additionally, because the rate of decline in T_CORE_ was the same in both saline and nor-BNI pretreatment conditions, the minimum T_CORE_ of 29.5 ± 0.8°C after CHA administration was reached at a shorter time (4 h:56 min ± 50 min, p = 0.0448) after the nor-BNI pretreatment than after the saline pretreatment. The nding that during the TI state in free-behaving rats^10^ pretreatment with nor-BNI reduced the maximum hypothermia but not the rate of decline in T_CORE_ suggests that Dyn, acting via central κ-opioid receptors, plays a permissive role in sustaining the skin cooling-induced inhibition of thermogenesis that is a hallmark of the TI state^13^.

## Discussion

Torpor/hibernation, naturally expressed by only a few species, is a complex behavioral phenotype^7^ with a unique metabolic state in which T_CORE_ and whole-body energy expenditure are markedly reduced through an inhibition of the normal, cold-defensive responses (BAT and shivering thermogenesis) that are essential for maintaining T_CORE_ in a cold environment^34^. We have discovered that a torpor-like state, featuring a shifted homeostasis closely resembling the physiological alterations observed in natural torpor, can be induced in rats^10, 13^, a species that does not naturally express torpor/hibernation in a cold environment. Further, the hypothermia and hypometabolism of this torpor-like state are due to a switch (Extended Fig. 3) within the CNS circuitry regulating body temperature, such that in the TI state stimulation of cold skin thermoreceptors produces an inhibition of thermogenesis^10, 13^. The present study reveals components of the principal afferent and efferent neural mechanisms underlying the TI state. We demonstrate that the well-known central thermoregulatory components, the PBN and the DMH^1–4, 6, 22^, mediate an inverted skin thermoreceptor regulation of thermogenesis during the TI state, which is independent from the integration of POA neuronal function required during normal thermoregulation. Our study also led to the discovery of a novel thermoregulatory function for a direct dynorphinergic pathway between the dlPBN and the DMH (Figs. 7, 10), playing an essential role in mediating the cold-evoked inhibition of thermogenesis that is a prominent characteristic of both the TI state and the torpor/hibernation state.

Neuraxis transection rostral to the DMH is sufficient to eliminate the normal, POA-dependent, skin thermoreceptor-mediated regulation of thermogenesis and to establish the TI state, in which thermogenesis is still controlled by skin thermoreceptors, but now responds to skin warming and cooling in an inverted^13^ way. This and our current nding that muscimol-induced inhibition of POA neurons also establishes the TI state are consistent with a model in which the switch from the normal to the TI thermoregulatory state requires a change in the activity of a population of POA neurons that significantly alters an input to the DMH. Recent studies to identify ‘torpor’ neurons in mice^8, 24, 25^ have also concluded that neurons in the POA are essential for establishing the hypothermic torpor state, including those Q neurons that project to the DMH^8^ and estrogen-sensitive neurons that project to medial hypothalamic areas^25^. However, none of these studies has proposed a neural circuit through which such ‘torpor neurons’ would regulate thermogenesis to induce hypothermia.

Paralleling the thermore ex circuit controlling BAT thermogenesis, cold-induced shivering is dependent on the activation of shivering thermogenesis-promoting neurons in the DMH that project to shivering premotor neurons in the rRPa^22^. Remarkably, the switch to the TI state of thermoregulation inverts the skin thermoreceptor-mediated regulation of shivering (Fig. 2), just as it does for BAT thermogenesis (Fig. 3). The warming-induced activation of BAT in the TI state requires an ionotropic glutamate receptor-mediated excitation of DMH neurons (Fig. 3), just as the cold-induced activation of BAT does in the normal thermoregulatory state^3^. Together, these ndings strongly support our conclusions that a modulation of inputs to neurons in the DMH is necessary to induce the TI state, and that the principal thermoregulatory mechanism underlying the cooling-induced hypothermia in the TI state is a widespread inhibition of thermogenesis at the level of the thermogenesis-promoting neurons in the DMH (Extended Data Fig. 2, 3).

Our demonstration that blockade of ionotropic glutamate receptors in the PBN prevents the warm-induced activation of BAT thermogenesis characteristic of the TI state indicates that ascending warm and cold thermoregulatory signaling, mediated by glutamatergic inputs to PBN neurons, is essential for the skin thermoreceptor-mediated control of thermogenesis in the TI state, as it is for normal thermoregulation^1^ in both the rat^3, 4^ and mouse^14^. Our discovery that the TI state occurs when the normal thermoregulatory connections between the POA and the DMH are severed (Fig. 3) or inhibited (Fig. 1), indicates the existence of POA-independent thermoregulatory pathways through which thermosensory signaling transmitted via PBN neurons can influence the activity of thermogenesis-promoting neurons in the DMH (Extended Data Fig. 3). Further, the inversion of the effects of cold and warm skin thermoreceptors on BAT and shivering thermogenesis in the TI state must arise from a “neuronal switch” that alters the balance between the skin thermoreceptor signaling that ascends from the PBN to the POA and the thermoreceptor signaling that short-circuits the POA to directly influence thermogenesis-promoting neurons in the DMH (Extended Data Fig. 3).

We identified connections between neurons in the dlPBN and elPBN regions of the PBN and neurons in the region of the DMH that contains thermogenesis-promoting neurons that project to the rRPa. Furthermore, we determined, in free-behaving and anesthetized naïve rats, that many of these DMH-projecting PBN neurons are active during normal thermoregulatory responses to skin cooling and skin warming. These ndings support the existence of novel thermoregulatory inputs from the PBN to the thermogenesis-promoting neurons in the DMH that could act in concert with those from the POA to contribute to the normal thermoregulatory control of thermogenesis. More specifically, we identified a population of DMH-projecting Dyn neurons in the dlPBN region that is activated during skin warming in naïve rats, when inhibitory influences on the discharge of thermogenesis-promoting neurons in DMH predominate. Dyn activated k-opioid receptor, has a potent inhibitory influence on thermogenesis-promoting neurons in the DMH, leading to a nearly complete reversal of the normal cold-evoked activation of BAT SNA and BAT thermogenesis (Fig. 8). These observations are consistent with a signi cant, but previously undescribed, thermogenesis-inhibiting role for these DMH-projecting Dyn neurons in the dlPBN during normal thermoregulation.

More Dyn neurons in the dlPBN were activated during skin cooling in the TI state, when thermogenesis is inhibited, than during skin cooling in naïve rats, when thermogenesis is active. In addition, blockade of the κ-opioid receptors for Dyn in the DMH markedly reduced the cold-evoked inhibition of BAT SNA in the TI state in anesthetized rats, and significantly reduced the maximum hypothermia induced in our torpor-mimicking CHA model of TI in awake rats^13^. Thus, Dyn acting via κ-opioid receptors in the DMH plays a necessary, permissive role in sustaining the skin cooling-induced inhibition of thermogenesis that is a hallmark of TI. However, it is important to note that activation of Dyn receptors in the DMH is not sufficient to induce the TI state. If the VGluT2-expressing Dyn neurons in dlPBN are glutamatergic, their role in inhibiting thermogenesis could be explained by their targeting of GABAergic interneurons in the DMH^35^. However, on the basis of the recent demonstration that the DMH axon terminals of VGluT2-expressing neurons in the POA express VGAT^15^, a marker for GABAergic terminals, we propose that the VGluT2-expressing Dyn neurons in the dlPBN might release both Dyn and GABA from their terminals in the DMH to provide a direct inhibitory regulation of thermogenesis-promoting neurons in the DMH. Together, these results provide strong support for our discovery of a novel thermoregulatory role for the dynorphinergic pathway from the dlPBN to the DMH in the inhibitory regulation of thermogenesis, both in the normal thermoregulatory state when skin warming inhibits thermogenesis, as well as in the TI state when skin cooling inhibits thermogenesis.

The fact that the neural circuitry underlying the TI state is present and functional in the rat, a species that does not express natural torpor, suggests that this neuronal substrate could be accessed for human clinical applications involving the management of hypothermia following ischemic incidents or for the reduction of metabolic demands during extended space ights. Further, the existence of an alternative thermoregulatory circuit mediating the TI state regulation of thermogenesis and energy expenditure could provide insight into the neural mechanisms underlying conditions in which thermoregulation (post-surgical shivering, anaphylactic or septic hypothermia) or energy metabolism (obesity) is regulated in a seemingly unphysiological manner.

## Material And Methods

### Animals

Male Sprague Dawley rats (300–400 g, Charles River Laboratories) were maintained in a standard 12 hr/12 hr, light/dark cycle (lights on at 0900) with ad libitum access to standard chow and water. Experiments were performed in accordance with the *Guide for the Care and Use of Laboratory Animals*, 8th Edition (National Research Council, National Academies Press, 2010) and protocols were approved by the Institutional Animal Care and Use Committee of Oregon Health and Science University.

### Procedures for recording BAT sympathetic nerve activity (SNA) or muscle shivering EMG

Rats were anesthetized initially with 3% isoflurane in 100% O_2_ and transitioned to urethane (0.8 g/kg) and chloralose (80mg/kg) following cannulation of a femoral artery and vein. Heart rate (HR) was derived from the femoral arterial pressure (AP) signal. Rats were positioned in a stereotaxic frame with the incisor bar at −4 mm below interaural zero and a spinal clamp installed on the T10 vertebra used to maintain the spine in a rigid and elevated position (detailed in ^13^). Rats were paralyzed with D-tubocurarine (0.3 mg initial dose, 0.1 mg/h supplements) and artificially ventilated with 100% O_2_ (60–70 cycles/min, tidal volume 3 – 3.5 ml). Thermocouples (Physitemp Instruments with Sable Systems International meter) were placed (a) on the shaved abdominal skin to measure the skin temperature (T_SKIN_) beneath a waterperfused blanket wrapped around the rat’s trunk, (b) 6 cm into the rectum to measure T_CORE_, and (c) into the medial aspect of the left interscapular BAT pad to measure BAT temperature (T_BAT_). T_CORE_ was normally maintained at ~ 37°C by perfusing the water blanket with warm water. As required for specific experimental protocols, T_SKIN_ and T_CORE_ were adjusted by changing the temperature of the water perfusing the thermal blanket.

Postganglionic BAT sympathetic nerve activity (SNA) was recorded from the central cut end of a small nerve bundle dissected from the ventral surface of the right interscapular BAT pad after dividing the fat pad along the midline and reflecting it laterally. BAT SNA was recorded with bipolar hook electrodes, ltered (1–300 Hz), and ampli ed (20,000x; Cyberamp 380, Axon Instruments). The viability and correct identification of the isolated BAT nerve were veri ed by signi cant increases in BAT SNA evoked by skin cooling.

A similar surgical preparation was used for experiments in which a shivering EMG was recorded with a bipolar electrode inserted into a nuchal (neck) muscle. Nuchal EMG (nEMG) recordings were performed in rats anesthetized initially with 3% isoflurane in 100% O2, and subsequently transitioned to a continuous intravenous infusion of inactin (85 mg/ml at 0.2 ml/h). Rats were arti cially ventilated but were not paralyzed.

### Pre-DMH transection (pre-DMH transX)

A cranial window (~ 4×4 mm) was made just behind the bregma and centered on the sagittal suture. The dura mater was carefully dissected from the superior sagittal sinus and removed throughout the cranial window to allow transection of the brain without damage to the sinus or the major vessels converging on it. A transection knife (15 mm long, 2 mm wide, and 0.1 mm thick) was mounted vertically in a stereotaxic manipulator and positioned perpendicular to the sagittal sinus at −1.5 mm caudal to bregma and with the medial edge on the midline. After a slight lateral retraction of the sagittal sinus, the knife was inserted into the brain sequentially on the left and right sides of the superior sagittal sinus to a depth of either − 9 mm (partial transection) or approximately − 10 mm (complete transection).

### Drug nanoinjection procedures

Intraparenchymal brain nanoinjections of drugs were performed as previously described^10, 16, 35^ via glass micropipettes, using a pressure injection system (Toohey model IIe). For repeated nanoinjections at the same site, the micropipette was retracted vertically, emptied, rinsed with saline, re lled, and repositioned at the original dorsoventral coordinate. The injection sites were marked with fluorescent polystyrene microspheres (1:10 dilution of FluoroSpheres F8797, F8801, or F8803, Invitrogen).

With the incisor bar positioned at −4 mm, bilateral nanoinjections (120 nl each) in the PBN were performed at −8.9 mm caudal to bregma, 2.2 mm from the midline, and 5.5 mm below the brain surface; and in the DMH at −3.2 mm caudal to bregma, 0.4 mm from the midline, and 7.8 mm below the brain surface. Bilateral nanoinjections (180 nl each) were performed bilaterally in the medial preoptic area (MPA; −0.4 mm caudal to bregma, ± 0.4 mm from the midline, and − 7.5 mm below the brain surface), and in the median preoptic area (MnPO; at bregma on the midline, −6.5 mm below the brain surface).

Following experimental procedures, rats were perfused transcardially with isotonic saline, followed by 4% paraformaldehyde (PFA) in 10 mM sodium phosphate buffered saline (PBS; pH 7.4). The brains were removed, post xed in 4% PFA (2 h), equilibrated overnight in 30% sucrose, and sectioned (60 μm coronal sections) to localize the fluorescent spots indicating the centers of the injection sites. The coordinates used for the brain intraparenchymal injections were adapted from a rat brain atlas ^36^ and from our previous studies involving these brain regions ^1, 13, 16^.

### Drugs

The A1 adenosine receptor (A1AR) agonist n6-cyclohexyladenosine (CHA, 1mM, Sigma Aldrich); the NMDA receptor antagonist (2R)-amino-5-phosphonopentanoate (AP5, 5 mM, Tocris); the AMPA/kainate receptor antagonist 6-cyano-7-nitroquinoxaline-2,3-dione disodium salt hydrate (CNQX, 5 mM, Tocris); the peptidase inhibitor (2S,3R)-3-Amino-2-hydroxy-5-methylhexanoyl-Val-Val-Asp hydrochloride hydrate (Amastatin, 1 mM, Sigma Aldrich); Dynorphin A (100 μM, Tocris); and the κ-opioid receptor antagonist nor-Binaltorphimine dihydrochloride (nor-BNI, 27 μM intraparenchymal or 1.6 mM ICV, Tocris) were dissolved in isotonic saline.

### Experiments in free-behaving rats

Central administration of the A1AR agonist, CHA, produces the TI state, featuring a dramatic fall in T_CORE_ due to an inhibition of thermogenesis in a cold T_AMB_^10^. Rats were anesthetized with 2% isoflurane in 100% O_2_ and instrumented for chronic recording of physiological variables as previously described ^10^. Rats were implanted with an intraperitoneal implantable temperature probe (Anipill^®^) for recording of T_CORE_. A guide cannula (C315G-26GA, PlasticsOne) was stereotaxically positioned in the lateral ventricle for intracerebroventricular (ICV) injection of drugs. The cannula was secured to the skull with screws and dental acrylic. Following the surgical procedure, rats were treated with buprenorphine (0.1 mg/kg), penicillin G (40,000 units/kg) and hydrated with isotonic saline (5 ml, subcutaneous). Each rat recovered for 7 days in a temperature-controlled recording chamber at an ambient temperature (T_AMB_) of 25°C and received daily meloxicam (1 mg/kg orally) for the rst 3 days to reduce post-surgical in ammation. For ICV injections of CHA and nor-BNI, 5 μl of drug solution were injected over 2 minutes through an internal cannula connected to a 25 μl Hamilton syringe. Rats were brie y removed from the recording chamber, the ICV injection procedure was performed in less than 5 minutes, and the rats were immediately returned to the recording chamber for continued data acquisition.

### Neuroanatomy

For anatomical tracing experiments, adult male Sprague Dawley rats (240–400 g) were anesthetized with 2–3% isoflurane in 100% O2, and stereotaxically injected with cholera toxin subunit b (CTb) conjugated with Alexa-488 (1 mg/ml, 120 nl) into the right DMH (bregma: 3.2 mm caudal, 0.4 mm lateral, 7.5 mm ventral to the brain surface; incisor bar at −4 mm), and FluoroGold (FG, 2%, 30 nl) into the rRPa (relative to lambda: 3.0 mm caudal, 0.0 mm lateral, 9.2 mm ventral to the brain surface; incisor bar at −4 mm). Rats were pretreated with intramuscular injections of an antibiotic (40,000 units/kg penicillin G) and an analgesic (1 mg/kg meloxicam), and subcutaneous injection of isotonic saline (3 ml). One week after tracer injections, free-behaving rats were exposed to a cold ambient (T_AMB_: 10°C) or to a warm ambient (T_AMB_: 30°C) for 2 h to elicit Fos expression as an indicator of neuronal activation. To maximize Fos expression, rats were maintained for 24 h at a T_AMB_ of 30°C prior to the 2 h cold exposure or maintained for 24 h at a T_AMB_ of 10°C prior to the 2 h warm exposure.

We employed retrograde transport, combined with Fos expression, to identify DMH-projecting neurons in PBN that are activated following induction of TI in anesthetized rats. Seven days prior to the terminal experiment, rats were injected with non-conjugated CTb into the right DMH as detailed above, with some rats also receiving an injection of FG into the rRPa to identify the DMH region containing thermogenesis-promoting neurons ^5, 35^. Following a 7-day recovery period for retrograde transport of CTb, rats were anesthetized with 2–3% isoflurane, placed in the stereotaxic frame, and prepared for pre-DMH transX surgery. Four groups were studied: (a) cold naïve (Cold-N) rats were maintained with a cold skin (T_SKIN_ < 35°C) for 1 h of baseline recordings with a stable T_SKIN_ and T_CORE_, after which they received a sham brain transection surgery (transection knife lowered into the cortex) and were then maintained with a cold skin for 2 h; (b) warm naïve (Warm-N) rats were treated similarly, but with a skin warming (T_SKIN_ > 35°C); (c) Cold-T rats were maintained with a cold skin for 1 h of baseline recording and then received a complete pre-DMH transX, followed by 2 h with a cold skin; (d) Warm-T rats were treated similarly, but with skin warming throughout the experiment. Following the sham or complete transX, rats were anesthetized with pentobarbital (80 mg/kg i.p.) and transcardially perfused with saline followed by 4% PFA. The brains were removed and post- xed in 4% PFA for 12 h and equilibrated overnight in 30% sucrose in PBS. Serial coronal sections (20 μm and 40 μm) were cut RNAse free in a microtome, collected sequentially in 6 sets (4 × 40 um, 2 × 20 um), and stored in cryoprotectant at −20°C.

An immuno fluorescence procedure was performed to label DMH-projecting PBN neurons and rRPa-projecting DMH neurons that express Fos in response to cold or warm exposure (from free-behaving and from anesthetized rats). Sections (40 μm) containing the PBN or the DMH were washed in PBS, blocked for 1 h in PBS with 0.3% Triton-X 100 (PBST) containing 3% normal donkey serum, and incubated overnight at room temperature in PBST containing the primary antibodies for Fos (rabbit anti-c-Fos, 1:2K; Encor, cat#: RPCA-c-Fos) and CTb (goat anti-CTb, 1:5K; Calbiochem, cat#: 227040). After several washes in PBS, sections were incubated for 2 h in PBST containing the secondary species-specific antibodies Alexa Fluor-594 donkey anti-rabbit IgG (1:500) and Alexa Fluor-488 donkey anti-goat IgG (1:250), both from Jackson ImmunoResearch. After incubation, sections were washed in PBS, mounted on Superfrost Plus slides, air-dried and coverslipped. DMH-projecting neurons in the PBN activated by cold or warm exposure displayed green cytoplasmic (CTb) and red nuclear (c-Fos) fluorescence. rRPa-projecting neurons in DMH (from rats injected with FG into the rRPa) displayed cytoplasmic FG and red nuclei when activated.

To identify the phenotypes of PBN neurons, mRNA transcripts for pro-Dynorphin (pDyn), vesicular glutamate transporter 2 (VGluT2), and vesicular GABA transporter (VGAT) were detected by in situ hybridization (ISH) using the RNAScope Multiplex Fluorescent v2 assay (Advanced Cell Diagnostics) in 20 μm-sections containing the PBN, following manufacturer instructions. mRNA transcripts were labeled with Opal uorophores (Akoya Biosciences): Opal-520 (1:500, green) for VGAT, Opal-570 (1:2K, red) for pDyn, and Opal-690 (1:500, far-red; assigned blue color in captured images) for VGluT2. Slides were coverslipped with anti-fade mounting medium (Pro-Long Gold, Invitrogen).

To characterize DMH-projecting (CTb-labeled) PBN neurons that express c-Fos in response to cold or warm exposure in the 4 groups of isoflurane-anesthetized rats, we used the RNA-Protein Co-detection ancillary kit (Advanced Cell Diagnostics). c-fos and pDyn mRNA transcripts were detected by ISH using the RNAScope procedure, whereas CTb was labeled with immuno fluorescence. Sections (20 μm) containing the PBN were rst incubated in the primary goat anti-CTb antibody (1:500) overnight at 4°C, then washed in PBST and subjected to the RNAscope Multiplex Fluorescent v2 assay following manufacturer instructions (with minor modi cations). mRNA transcripts were labeled with Opal uorophores (Opal-570, red) and (Opal-690, far-red assigned blue color). After RNAScope procedure, slides were washed in PBST and incubated for 2 h in the species-specific secondary antibody for CTb (Alexa-488 donkey anti-goat, 1:200). Slides were washed in PBST and coverslipped with anti-fade mounting medium. CTb-labeled neurons displayed cytoplasmic green fluorescence, whereas c-fos and pDyn mRNA transcripts were labeled as red and far-red (blue) punctate, respectively. In some cases, the colors were reversed for c-fos and pDyn transcripts.

All slides processed for IHC and ISH were visualized at an Olympus BX-51 fluorescence microscope, and images were captured using Simple PCI software (C-Imaging Systems). Brightness and contrast were adjusted using Adobe Photoshop.

The CTb antigenicity is severely impaired by the protease step of the ISH procedure, causing a substantial decrease in CTb labeling. In addition, the pDyn and c-fos transcript signals are very strong; therefore, the number of triple-labeled neurons (CTb-pDyn-c-fos) was significantly underestimated with this co-detection procedure.

To further visualize Dyn-containing neurons in the PBN, a group of rats (n = 3) were injected, after 7 days recovery from previous injection of CTb in DMH, by an ICV injection of colchicine, which disrupts axonal transport and concentrates the neuropeptide in the soma. Rats were injected, under general anesthesia, with 10 μl of colchicine (25 mM in 10% DMSO saline) ICV. Rats were allowed to survive for 24–36 h after colchicine injection and were perfused with PFA. Brains were removed and processed for IHC as described above.

### Data acquisition

Physiological variables were digitized (Micro 1401 MKII; Cambridge Electronic Design) at the following rates: BAT SNA (1 kHz), T_BAT_ (5 Hz), T_CORE_ (5 Hz), T_SKIN_ (5 Hz), T_PAW_ (5 Hz), expired CO_2_ (200 Hz), AP (200 Hz), EKG (10–300 Hz, 1 kHz), EMG (10–300 Hz, 5 kHz) and recorded into a computer hard drive for subsequent analysis (Spike 2, CED). Continuous measures (4 s bins) of BAT SNA and nEMG amplitudes were calculated as the root mean square (rms, square root of the total power in the 0.1 to 20 Hz band for BAT SNA, and in the 0.1–500 Hz band for EMG) value of sequential 4-s segments of the BAT SNA and nEMG signals ^13, 22^.

### Data and statistical analysis

For analysis of the physiological variables, the data were averaged into 30s bins, and group data were reported as mean ± standard error of the mean (SEM). To account for slight differences in BAT SNA and nEMG recording characteristics (e.g., tissue-electrode contact, ampli er noise, etc.) among experiments, values for BAT SNA and nEMG throughout each experiment were expressed as ‘percent baseline’ (%BL), where baseline values for BAT SNA and for nEMG were the low levels recorded under warm T_SKIN_ and warm T_CORE_ conditions when BAT SNA and nEMG are at their minimum levels in naïve rats. Treatment effects on BAT SNA or nEMG are quanti ed as the difference between pre-treatment (control) and post-treatment levels of BAT SNA and in nEMG.

For analysis of anatomical data, neurons expressing Fos, CTb, and CTbFos were counted in the PBN in the side ipsilateral to the CTb injection in DMH. Counting was performed in the dlPBN and elPBN in 4 sections (separated 100 μm) that were labeled as “rostral” (Bregma: −8.90 mm), “intermediate-1” (Bregma: −9.00 mm), “intermediate-2” (Bregma: −9.10 mm), and “caudal” (Bregma: −9.20 mm), based on the Paxinos rat brain atlas ^36^. Counts at each rostro-caudal level or total counts (sum of counts from the 4 levels) were used in different analysis. The counts of double-labeled (CTbFos) neurons were normalized to the number of CTb-labeled neurons (% CTbFos/CTb) to counteract variability among CTb injections.

Neurons expressing Fos, FG, and FGFos were counted in one DMH hemi-section, located contralateral to the CTb injection in DMH, at the level where the main FG-ir cluster is located (i.e., neurons projecting to the rRPa). The counts of double-labeled (FGFos) neurons were normalized to the number of FG-labeled neurons (% FGFos/FG) to counteract variability among FG injections.

Data are reported as mean ± SEM. All statistics were performed using Prism software (version 6, GraphPad Software Inc.). Paired one- or two-tailed t-tests, one-way ANOVA followed by t-test, and a two-way ANOVA followed by post-hoc Bonferroni correction were used for statistical comparisons, as described in figure legends. Statistical results with p < 0.05 were considered significant.

## Data Availability

All data needed to evaluate the conclusions in the paper are present in the paper and/or the Supplementary Materials.
